# Noninvasive assessment of endometrial fibrosis in patients with intravoxel incoherent motion MR imaging

**DOI:** 10.1038/s41598-021-92383-w

**Published:** 2021-06-18

**Authors:** Qing Hu, Peipei Jiang, Yongjing Feng, Yan Xu, Nan Zhou, Weibo Chen, Li Zhu, Yali Hu, Zhengyang Zhou

**Affiliations:** 1 Department of Radiology, Nanjing Drum Tower Hospital, The Affiliated Hospital of Nanjing University Medical School, Nanjing, 210008 China; 2 Department of Obstetrics and Gynecology, Nanjing Drum Tower Hospital, The Affiliated Hospital of Nanjing University Medical School, Nanjing, 210008 China; 3 Philips Healthcare, Shanghai, 200233 China

**Keywords:** Diagnostic markers, Reproductive disorders, Diagnosis, Medical imaging

## Abstract

Recently, few noninvasive methods have been reported to evaluate endometrial fibrosis. Our study was to investigate the feasibility of intravoxel incoherent motion (IVIM) MR imaging in the detection of endometrial fibrosis in patients with intrauterine injury. 30 patients with hysteroscopy-confirmed endometrial fibrosis and 28 healthy women were enrolled to undergo MR examination including the IVIM sequence. Endometrial thickness (ET); apparent diffusion coefficient (ADC); and IVIM parameters, including pure diffusion coefficient (*D*), pseudodiffusion coefficient (D*) and vascular fraction (*f*) were evaluated. A multivariable model combing ADC, *D*, and *f* values using binary logistic regression analysis was built to diagnose endometrial fibrosis. Endometrial fibrosis patients demonstrated lower endometrial ADC, *D*, *f* values and ET (all p < 0.05). The multivariable model, ADC, *D*, *f* values and ET performed well in diagnosing endometrial fibrosis with AUC of 0.979, 0.965, 0.920, 0.901 and 0.833, respectively*.* The multivariable model revealed a better diagnostic accuracy than *D*, *f* and ET (all p < 0.05). Although ADC achieved a better diagnostic value than ET (z = 2.082, p < 0.05), no difference in AUC was shown among ADC, *D*, and *f* (all p > 0.05); between ET and *D* (p > 0.05); and between ET and *f* (p > 0.05). The reproducibility of ADC, *D*, *f* and D* values in patients with endometrial fibrosis and healthy women were good to excellent (ICC: 0.614–0.951). IVIM parameters exhibit promising potential to serve as imaging biomarkers in the noninvasive assessment of endometrial fibrosis.

## Introduction

Endometrial fibrosis is a repair process of the endometrium that is commonly associated with trauma to the endometrium from surgical procedures, primarily curettage^1,2^. Histopathologically, the fibrotic endometrium is characterized by poor epithelial growth, poor vascular development and the displacement of extracellular matrix by the fibrous connective tissues, leading to embryo implantation dysfunction and consequent infertility or spontaneous abortion^1,3,4^. Timely diagnosis of endometrial fibrosis could promote antifibrotic treatment with regenerative medicine and prevent further development of intrauterine adhesions^3^. Currently, hysteroscopy is considered the gold standard for the diagnosis of endometrial fibrosis, but it is an invasive and painful procedure with a risk of secondary injury of the endometrium^5,6^. In addition, it is certainly not the ideal procedure for serially repeated assessment of fibrotic progression. Hence, noninvasive and reliable imaging modalities are optimal alternatives for the diagnosis and assessment of endometrial fibrosis.

Given its noninvasive nature and universal availability, ultrasonography (US) is the modality most often used for the assessment of endometrial fibrosis. US can reveal morphological abnormalities, such as distinctly thinner endometrium and irregular interruptions in the lining at the sites of fibrosis^1,7^. Due to excellent soft tissue contrast and high spatial resolution, MR imaging may demonstrate a partial or complete absence of the normal high signal endometrial layer in patients with endometrial fibrosis on T2-weighted images^8^. However, these conventional imaging techniques cannot quantitatively assess the degree of fibrosis, reflect the biological abnormality of endometrial fibrosis at the cellular level, and evaluate the function of the remaining endometrium.

As a quantitative imaging technique, intravoxel incoherent motion (IVIM) MR imaging can be used to noninvasively investigate diffusivity and microcapillary perfusion in biological tissues^9,10^. In recent years, it has been successfully used to stage liver fibrosis, to evaluate the degree of fibrosis of the kidney and pancreas, and to detect parotid gland and intestinal fibrosis^11–15^. Based on those studies and the histopathology of endometrial fibrosis, it is presumable that IVIM MR imaging might serve as a potential imaging biomarker for evaluating endometrial fibrosis.

The purpose of this study was to investigate the difference in IVIM-derived parameters of the endometrium between patients with endometrial fibrosis and healthy women and to explore the diagnostic performance of IVIM parameters in endometrial fibrosis.

## Results

### Study population

**Figure 1:**
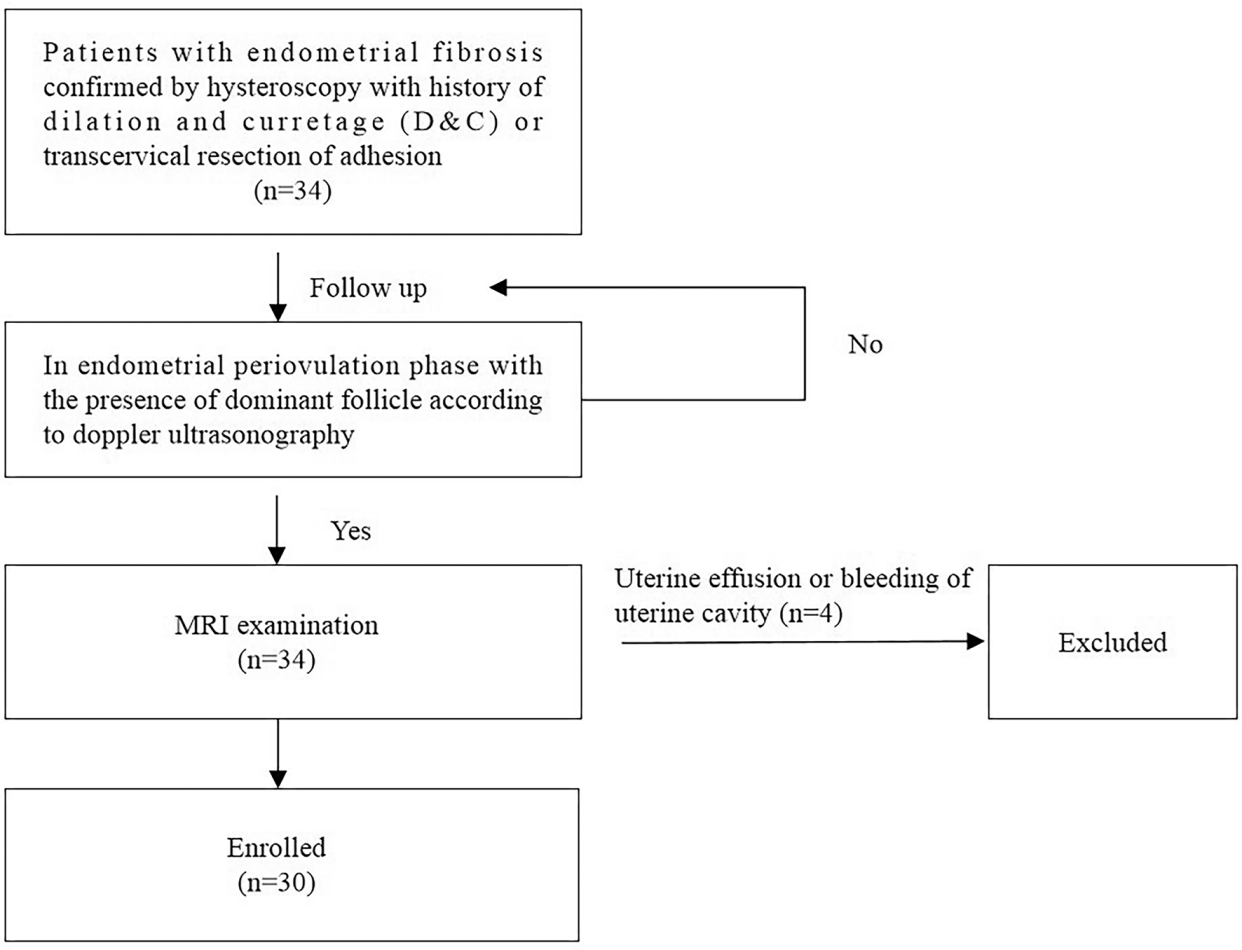
A flow chart of the enrolled patients.

All the patients underwent dilation and curettage (D&C) once or several times, and some of them experienced transcervical resection of adhesions. The clinical demographics of patients and healthy women are detailed in Table 1. The process of patient inclusion and exclusion is displayed in a flow chart (Fig. 1). Four patients and one healthy woman were excluded for poor image quality caused by massive uterine effusion or bleeding of the uterine cavity. Finally, 30 patients (mean age: 33.30 years; range 27–42 years) and 28 healthy women (mean age: 29.00 years; range 24–38 years) were included in this study.

**Table 1:** The clinical demographics of patients with endometrial fibrosis and healthy women.

Clinical features	Patients	Healthy women
Number	30	28
Age (years)	33.3 (27–42)	29.0 (24–38)
**Concomitant uterus conditions**		
No	13 (43.3%)	28 (100%)
Cervical cyst	7 (23.3%)	0 (0%)
Uterine cyst	1 (3.3%)	0 (0%)
Both cervical and uterine cyst	9 (30%)	0 (0%)
**Number of D&C**		
1	9 (30.0%)	0 (0%)
2	9 (30.0%)	0 (0%)
≥ 3	12 (40%)	0 (0%)
**Transcervical resection of adhesion**		
Yes	23 (76.7%)	0 (0%)
No	7 (23.3%)	0 (0%)

Data are number (percentage) or mean (range).

*D&C* dilation and curettage.

### Differences in IVIM parameter values and ET between patients and healthy women

**Figure 2:**
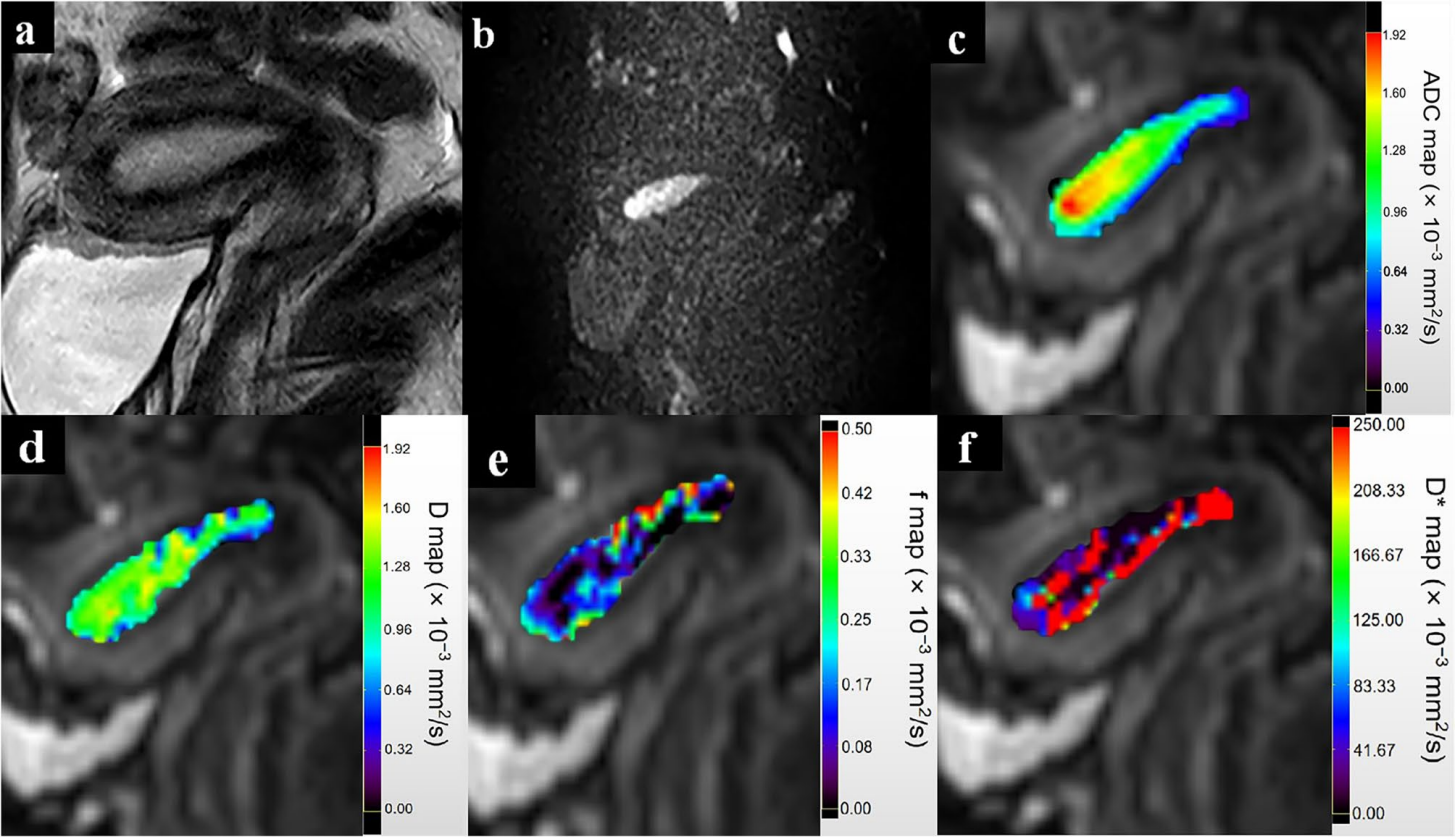
A 24-year-old healthy woman. (**a**) Sagittal T2-weighted turbo spin-echo (TSE) image demonstrates the long ribbon shape normal endometrium with high signal intensity. (**b**) Sagittal diffusion-weighted image (b = 800 s/mm^2^) of the uterus. Parametric maps of ADC (**c**), *D* (**d**), *f* (**e**) and D* (**f**) demonstrate that the average ADC, *D*, *f* and D* values of the normal endometrium are 1.51 × 10^−3^ mm^2^/s, 1.36 × 10^−3^ mm^2^/s, 0.12 and 66.82 × 10^−3^ mm^2^/s, respectively. *ADC* apparent diffusion coefficient, *D* true diffusion coefficient, *f* perfusion fraction, D* pseudodiffusion coefficient.

**Figure 3:**
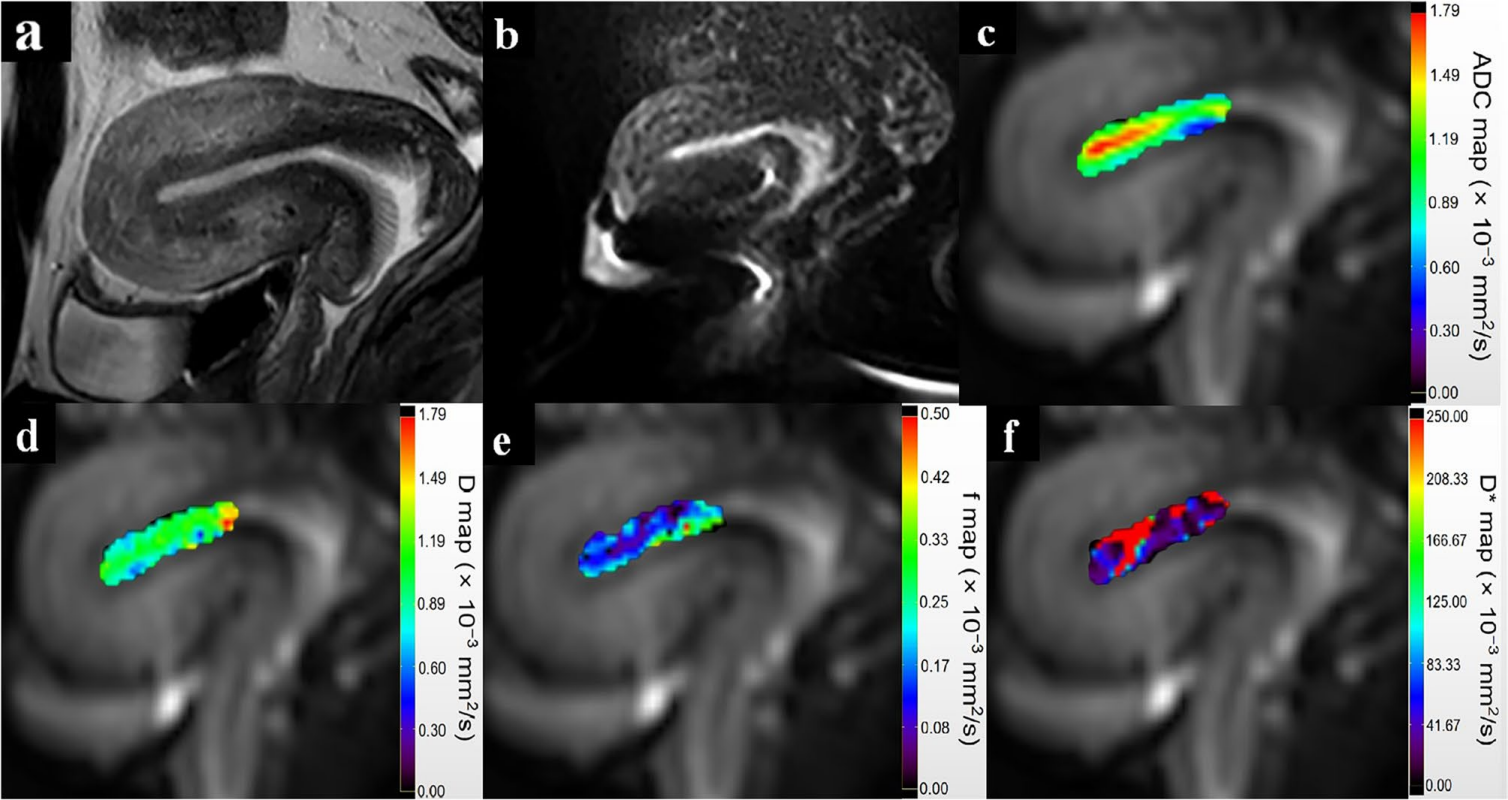
A 33-year-old patient with hysteroscopy confirmed endometrial fibrosis. (**a**) Sagittal T2-weighted turbo spin-echo (TSE) image reveals a thin ribbon-shaped endometrium with multiple cysts in the cervix. (**b**) Sagittal diffusion-weighted image (b = 800 s/mm^2^) of the uterus depicts the high signal intensity endometrium. Parametric maps of ADC (**c**), *D* (**d**), *f* (e) and D* (**f**) demonstrate that the average ADC, *D*, *f* and D* values of the fibrous endometrium are 1.0 × 10^−3^ mm^2^/s, 0.90 × 10^−3^ mm^2^/s, 0.10 and 34.46 × 10^−3^ mm^2^/s, respectively. *ADC* apparent diffusion coefficient, *D* true diffusion coefficient, *f* perfusion fraction, D* pseudodiffusion coefficient.

As shown in Table 2, the mean ADC, *D*, *f* values and ET was significantly lower in patients with endometrial fibrosis (all p < 0.05). However, no significant difference in the D* value was noted between patients and healthy women (p > 0.05). Figures 2 and 3 show T2WI, DWI and the corresponding parametric maps (ADC, *D*, *f* and D* maps) of the uterus in a healthy woman and a patient.

**Table 2:** Differences of IVIM parameters values and ET between patients and healthy women.

Parameters	Patients	Healthy volunteers	P values
ADC (× 10^−3^ mm^2^/s)	1.14 ± 0.09 (1.15, 1.07–1.20)	1.43 ± 0.15 (1.44, 1.30–1.53)	< 0.05*
*D* (× 10^−3^ mm^2^/s)	1.03 ± 0.09 (1.02, 0.97–1.10)	1.23 ± 0.10 (1.24, 1.16–1.32)	< 0.05*
*f*	0.09 ± 0.02 (0.10, 0.07–0.10)	0.14 ± 0.04 (0.13, 0.11–0.15)	< 0.05*
D* (× 10^−3^ mm^2^/s)	36.28 ± 24.53 (27.45, 19.57–47.30)	48.11 ± 37.92 (33.81, 19.06–64.13)	0.40
ET (mm)	8.39 ± 0.95 (8.26, 7.75–9.09)	11.10 ± 2.60 (11.27, 9.31–12.30)	< 0.05*

Data are mean ± standard deviation (median, interquartile range).

*ADC* apparent diffusion coefficient, *D* true diffusion coefficient, *f* perfusion fraction, D* pseudo-diffusion coefficient, *ET* endometrial thickness.

*p < 0.05 with independent sample T test or Mann–Whitney U test.

### Performance of IVIM parameters, ET and the multivariable model of ADC, D and f values in diagnosing endometrial fibrosis

**Figure 4:**
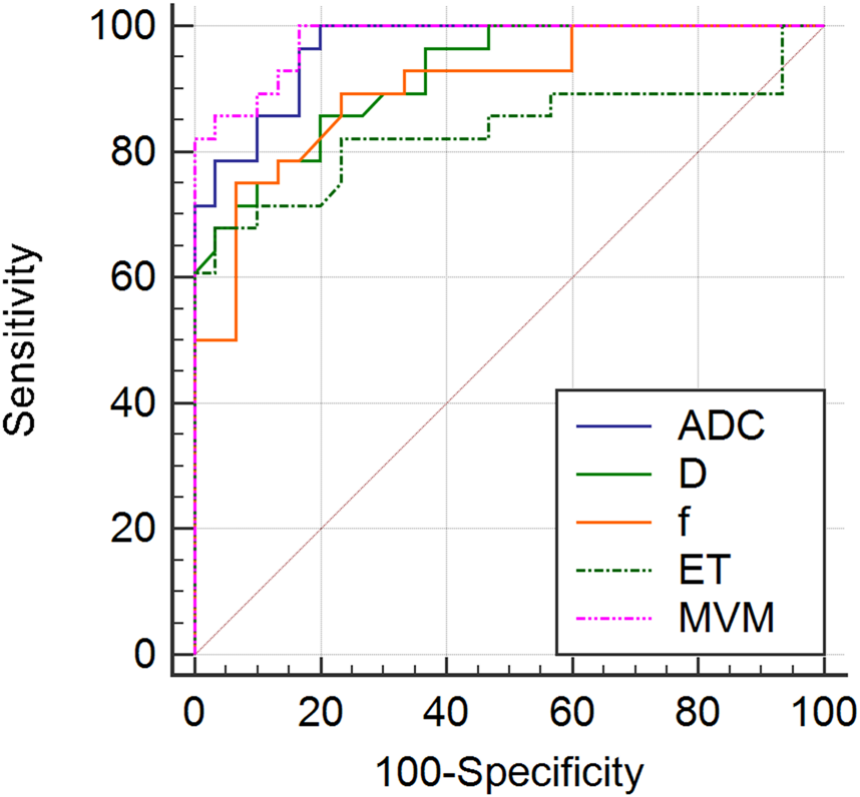
ROC curves for the performance of ADC, *D*, *f* values, ET and the multivariable model of ADC, *D* and *f* values for distinguishing patients with endometrial fibrosis and healthy women. *ROC* receiver operating characteristic, *ADC* apparent diffusion coefficient, *AUC* area under ROC curve, *D* true diffusion coefficient, *f* perfusion fraction, *ET* endometrial thickness, *MVM* a multivariable model combing ADC, *D* and *f* values using the binary logistic regression analysis.

As shown in Table 3, the performance of the multivariable model, ADC, *D*, *f* values and ET in diagnosing endometrial fibrosis were all excellent with AUCs of 0.979, 0.965, 0.920, 0.901, and 0.833, respectively, and the multivariable model had the highest AUC. Figure 4 shows the ROC curves of the multivariable model, ADC, *D*, *f* values and ET for distinguishing patients with endometrial fibrosis from healthy women. The multivariable model revealed a better diagnostic performance than *D*, *f* and ET (z = 1.980, 2.190, 2.406, respectively, all p < 0.05). ADC achieved a better diagnostic value than ET (z = 2.082, p < 0.05). No difference in AUC was shown between the multivariable model and ADC (p > 0.05), among ADC, *D*, and *f* (all p > 0.05); between ET and *D* (p > 0.05); and between ET and *f* (p > 0.05). The false negative rates for ADC, *D* and *f* values in the diagnosis of endometrial fibrosis were 20%, 20%, and 25%, respectively. The false positive rates for ADC, *D* and *f* values in the diagnosis of endometrial fibrosis were 0%, 14.3%, and 6.7%, respectively.

**Table 3:** Performance of ADC, *D*, *f*, ET and the multivariable model of ADC, *D* and *f* values in diagnosing endometrial fibrosis.

Parameters	AUC (95% CI)	Sensitivity (%)*	Specificity (%)*	Cut-off value
ADC (× 10^−3^ mm^2^/s)	0.965 (0.881–0.996)	80.00 (24/30)	100 (28/28)	1.21
*D* (× 10^−3^ mm^2^/s)	0.920 (0.819–0.975)	80.00 (24/30)	85.71 (24/28)	1.10
*f*	0.901 (0.794–0.964)	93.33 (28/30)	75.00 (21/28)	0.11
ET (mm)	0.833 (0.712–0.918)	96.67 (29/30)	67.86 (19/28)	9.65
MVM	0.979 (0.901–0.999)	83.33 (25/30)	100 (28/28)	0.75

*ADC* apparent diffusion coefficient, *D* true diffusion coefficient, *f* perfusion fraction, *ET* endometrial thickness, *MVM* a multivariable model combing ADC, *D* and *f* values using the binary logistic regression analysis, *AUC* area under ROC curve, *CI* confidence interval.

*Data in parenthesis are numerator/denominator.

### Reproducibility in the measurements of IVIM parameters

As shown in Table 4, the intra- and interobserver agreements in the measurements of ADC and *D* values in patients with endometrial fibrosis and healthy women were excellent (all ICC > 0.800); for the measurements of *f* and D* values, the intra- and interobserver agreements were good to excellent (ICC: 0.614–0.935).

**Table 4:** Intra- and inter-observer agreements in the measurements of IVIM parameters of endometrium in patients and healthy women.

Parameters	Intra-observer ICC (95% CI)		Inter-observer ICC (95% CI)	
	Patients	Healthy women	Patients	Healthy women
ADC (× 10^−3^ mm^2^/s)	0.883 (0.754–0.994)	0.951 (0.894–0.977)	0.858 (0.702–0.933)	0.878 (0.737–0.944)
*D* (× 10^−3^ mm^2^/s)	0.878 (0.744–0.942)	0.923 (0.834–0.964)	0.838 (0.660–0.923)	0.874 (0.727–0.942)
*f*	0.791 (0.560–0.900)	0.671 (0.290–0.848)	0.935 (0.864–0.969)	0.792 (0.551–0.904)
D* (× 10^−3^ mm^2^/s)	0.614 (0.189–0.816)	0.876 (0.732–0.943)	0.812 (0.606–0.911)	0.795 (0.556–0.905)

*ADC* apparent diffusion coefficient, *D* true diffusion coefficient, *f* perfusion fraction, D* pseudo-diffusion coefficient, *ICC* intraclass correlation coefficient, *CI* confidence interval.

## Discussion

Our pilot study demonstrated differences in IVIM parameter values and ET between patients with endometrial fibrosis and healthy women. In addition, endometrial ADC, *D*, *f* values, ET and the multivariable model had good efficiency for diagnosing endometrial fibrosis. The intraobserver and interobserver agreements of endometrial ADC, *D* and *f* value measurements were good.

In the present study, we observed that ADCs and diffusion-linked component *D* values decreased significantly in the fibrous endometrium. This finding could be explained by the fact that normal endometrial glands and stromal cells could become atrophic and replaced by simple cuboidal epithelium incrementally as the fibrosis of endometrium progressed^1,16^. The subsequent denser cellularity and abundant accumulation of extracellular matrix (ECM), especially fibrillary collagens, could inhibit the random motion of water molecules, which might result in the reduction of ADC and *D* values^3,10,16^. Similar to other organ fibrosis, due to the presence of endometrium perfusion rather than pure diffusion restrictions, ADC values were greater than the corresponding *D* values^10,15,17^. Thus, compared with the biexponential model, a monoexponential model could overestimate the water diffusion in the fibrotic tissue.

According to previous studies, it is widely held that blood perfusion decreases in fibrotic tissue due to concomitant alterations in tissue microcirculation, including damage to capillary networks, proliferation of connective tissue and increased resistance to blood flow^10,15^. This might explain the lower *f* value in fibrotic endometrium compared with normal endometrium in this study. The decreased blood perfusion might lead to embryo implantation dysfunction and consequent infertility or spontaneous abortion^1,3^. Thus, we hypothesize that the *f* value may have the potential to serve as an imaging biomarker reflecting endometrium perfusion without the administration of contrast agents.

Interestingly, in this study, the perfusion-related parameter D* value exhibited no difference between patients and healthy women. Generally, the D* value could reflect endovascular blood flow velocity within tissue, which was significantly lower in fibrotic organs^16,18,19^. However, D* value is well known for its large standard deviation, data instability and its dependence on signal-to-noise ratio (SNR)^14,19,20^, which limits its clinical applicability. Further studies are warranted to focus on these deficiencies and improve the veracity of D*.

Furthermore, it is worth noting that all of the participants in this study underwent MR examination during the periovulation phase due to the existence of the physiological dynamic changes in endometrial thickness and microstructure over the different phases of the menstrual cycle^21^.

Previous studies confirmed that trauma to the basal layer of the endometrium caused endometrial fibrosis and affected the regeneration of endometrial epithelial cells, which led to a thin endometrium^1^. Although thin endometrium could be used as one of the predictive markers of endometrial fibrosis, it does not mean that all thin endometriums are from patients with endometrial fibrosis. In other words, among women with thin endometrium, only those whose endometrium has abnormal microstructure and function are diagnosed with endometrial fibrosis. It has been reported that some women with endometrium thinner than 4 or 5 mm may still have normal endometrial function and become pregnant successfully^22,23^. Thus, in this study, IVIM-DWI as a functional imaging technique demonstrated superior capability to conventional MR imaging according to reflecting the biologic abnormality of the body at a cellular level^9,10^.

This study initially revealed the feasibility of IVIM MR imaging for the evaluation of patients with endometrial fibrosis. Significantly lower ADC, *D* and *f* values were detected in the endometrium of patients with endometrial fibrosis. Though ADC showed higher AUC compared to *D* and *f*, no significant differences were found. Moreover, the IVIM parameter *f* could reflect the perfusion changes in endometrial fibrosis^9^, which cannot be obtained with the conventional DWI model. So we insisted that compared with DWI, IVIM parameters could provide added value on the evaluation of the perfusion changes in fibrotic endometrium. The multivariable model showed the highest diagnostic performance in endometrial fibrosis, which indicated that the multivariable model might provide better diagnostic performance than the single IVIM parameter. There were relative low false negative/positives for ADC, *D* and *f* values in the diagnosis of endometrial fibrosis. Not all methods fail on the same patient, and the IVIM parameters are complementary in diagnosing endometrial fibrosis. In addition, ADC, *D* and *f* values exhibited good intraobserver and interobserver agreements. In a word, this study revealed that both the multivariable model and IVIM parameters had promising potential in the diagnosis of endometrial fibrosis, which might be helpful for clinicians to implement antifibrotic therapy, and to conduct dynamic follow-ups.

There are several limitations in this study. First, the sample size was relatively small, and patients with early endometrial fibrosis were absent. Nevertheless, the feasibility of utilizing quantitative MR imaging, including IVIM parameters and ET, to evaluate endometrial fibrosis was demonstrated. Second, due to the small sample size, we were unable to further stratify grades of endometrial fibrosis according to the IVIM parameters. Therefore, in future studies, a large cohort of patients will be recruited. Third, as the appropriate number of b values suitable for endometrial fibrosis remains unknown, further investigation is required.

In conclusion, our study confirmed the significant differences in intravoxel incoherent motion (IVIM) derived parameters of the endometrium between patients with endometrial fibrosis and healthy women. IVIM parameters provided functional features of the fibrotic endometrium, in which ADC, *D* and *f* values performed well in differentiating fibrotic endometrium from normal endometrium. It is conceivable that IVIM MR imaging has the potential to serve as an imaging biomarker in the noninvasive diagnosis of endometrial fibrosis.

## Materials and methods

### Subjects

This prospective study was approved by the ethics committee of the Institutional Review Board of Nanjing Drum Tower Hospital, and written informed consent was obtained from all participants. All experiments were performed in accordance with relevant guidelines and regulations. From October 2018 to December 2019, 34 patients with endometrial fibrosis confirmed by hysteroscopy (mean age: 33.56 years; range 27–42 years) and 29 healthy women with normal endometrium who served as the controls (mean age: 29 years; range 24–38 years) were recruited consecutively in our study.

The inclusion criteria for patients were as follows: (1) clinically diagnosed as infertile women with a history of intrauterine surgery, such as D&C or transcervical resection of adhesion; (2) endometrial scars confirmed by hysteroscopy; (3) no history of other severe uterine diseases, including adenomyosis, large intramural myomas, endometrial tuberculosis and severe congenital uterine malformations; (4) normal ovarian function; and (5) no MRI contraindications, such as cardiac pacemakers, cochlear implants and claustrophobia.

The criteria for inclusion in the healthy women group were as follows: (1) women of reproductive age with regular menstrual cycle and normal menstrual volume; (2) no history of uterine diseases such as uterine malformation, uterine tumors and intrauterine infection; (3) no history of abortion, D&C, or other uterine injuries; and (4) no MRI contraindications.

### MR acquisition

All MR imaging examinations were performed on a 3-T MR Philips scanner (Ingenia, Philips Medical Systems, Best, The Netherlands) with a 16-channel torso phased-array body coil. Before MR examinations, all participants were confirmed in their endometrial periovulation phase with the presence of dominant follicles according to Doppler ultrasonography. All participants were placed in a supine position with head-first and were asked to keep their abdomen as still as possible with free breath during the examination time. MRI protocols included sagittal T2-weighted turbo spin-echo (repetition time [TR] = 3927 ms, echo time [TE] = 100 ms, matrix size = 200 × 166, field of view = 120 mm × 120 mm, slice thickness = 3 mm, intersection gap = 0.3 mm, and number of signal averages [NSA] = 1.1) and axial T2-weighted turbo spin-echo (TR = 3659 ms, TE = 100 ms, matrix size = 120 × 64, field of view = 240 mm × 240 mm, slice thickness = 4 mm, intersection gap = 1 mm, and NSA = 1).

The IVIM sequences were obtained with 9 b values (0, 25, 50, 75, 100, 150, 200, 500, and 800 s/mm^2^) based on sagittal single-shot echo-planar imaging (SS-EPI) (TR = 3080 ms, TE = 53 ms, matrix size = 64 × 75, field of view = 160 mm × 160 mm, slice thickness = 3 mm, intersection gap = 0.5 mm, and NSA = 2). The scanning time of IVIM was approximately 2 min 37 s, and the total scanning time was approximately 8 min 51 s. All participants underwent the examinations successfully without any discomfort or side effects.

### MR analysis

MR images were independently reviewed and analyzed by two radiologists (Li Zhu and Zhengyang Zhou, with 6 and 15 years of experience in gynecology radiology, respectively) who were blinded to the clinical information of the patients. The final results of one participant were calculated from averaged values of the two radiologists. IVIM data were repeatedly measured by the first radiologist one month later for intra- and interobserver reproducibility analyses.

All MR images were transferred to the Picture Achieving and Communication System (PACS) Workstation. Endometrial thickness (ET) was measured on midsagittal T2-weighted images.

The IVIM data were evaluated using DWI-Tool developed by Philips in IDL 6.3 (ITT Visual Information Solutions, Boulder, CO, USA), and the *D*, D*, and *f* maps were generated automatically. The quantitative values of *D*, D* and* f* were calculated using the biexponential model raised by Le Bihan^9^ with the following equation:

1 $$ Sb/S0 = (1 - f) \cdot \exp ( - bD) + f \cdot \exp \left[ { - b \cdot (D + D*)} \right], $$

in which Sb and S0 denote the mean signal intensity at a specific b value and when the b value is 0 s/mm^2^, respectively. The ADC maps and the corresponding values were estimated by using the monoexponential fit function at all 9 b values:

2 $$ In(Sb) = In(S0) - bADC. $$

The specific slice of DWI with the largest endometrial section was selected referring to the corresponding sagittal T2-weighted images. Then, a region of interest (ROI) was manually drawn to include as much endometrium as possible. The ROI was positioned carefully inside the contour of the endometrium in the upper 2/3 uterus corpus and delineated on a relatively homogeneous endometrial region to avoid the areas with large degree of heterogeneity in the parametric maps. The ROIs were automatically propagated between the mono- and biexponential models to produce the corresponding *D*, D*, *f* and ADC values. Three ROIs were drawn repeatedly by each radiologist, and the average value of a total of six ROIs served as the representative result for subsequent statistical analysis.

### Statistical analysis

Statistical analysis was performed with SPSS 22.0 (SPSS Inc., IL, USA). Continuous variables with a normal distribution are presented as the means ± standard deviations (SD), whereas nonnormally distributed variables are presented as the median or interquartile range. Independent t tests were used to compare the differences in ET, ADC and *D* values between patients and healthy women. For the comparison of D* and* f* values obtained from patients and healthy women, the Mann–Whitney U test was used. A multivariable model combing ADC, *D*, and *f* values using the binary logistic regression analysis was built to diagnose endometrial fibrosis. Receiver operating characteristic (ROC) curve analysis was performed to evaluate the diagnostic performance of ADC, *D*, *f*, ET and the multivariable model for the discrimination of endometrial fibrosis from normal endometrium using the DeLong test in MedCalc 19.1.0.0 (MedCalc statistical software, Mariakerke, Belgium). The area under the curve (AUC) and the optimal cutoff value of each parameter for achieving the best diagnostic accuracy were calculated using the De Long test, and an AUC greater than 0.80 defined as excellent diagnostic efficacy. Additionally, intra- and interobserver reproducibility were evaluated using the intraclass correlation coefficient (ICC), which was classified as excellent ( 0.81–1.00), good ( 0.61–0.80), moderate (0.41–0.60), fair (0.21–0.40), and poor (0.00–0.20)^24^. p-values less than 0.05 were considered statistically significant.

## Data Availability

The datasets generated during and/or analysed during the current study are available from the corresponding author on reasonable request.
